# A discussion on the implementation of the Polar Code and the STCW Convention’s training requirements for ice navigation in polar waters

**DOI:** 10.1007/s12198-021-00241-7

**Published:** 2022-04-12

**Authors:** Espen Engtrø

**Affiliations:** Faculty of Science and Technology, University of Stavanger, Stavanger, Norway

**Keywords:** Polar Code, STCW, Training requirements, STAMP, Risk management

## Abstract

In 2017, the International Maritime Organization (IMO) implemented the *International Code for Ships Operating in Polar Waters* (Polar Code), with mandatory requirements covering the Arctic and Antarctic Oceans. In this conjunction, the *International Convention on Standards of Training, Certification and Watchkeeping* (STCW) were amended in 2018. New training requirements were made applicable for dedicated personnel in charge of a navigational watch on ships with a Polar Ship Certificate (PSC) operating in polar waters. In association with the new training requirements amending the STCW Convention, the IMO, and Transport Canada (flag state authority) signed a Memorandum of Understanding in 2017, for Canada to develop and deliver four regional capacity-building “train-the-trainer” workshops. The objectives of these events were to assist maritime education and training (MET) institutes in enhancing the skills and competence of instructors, to develop competence-based STCW training programs, for dedicated personnel on ships operating in polar waters. This paper examines the first workshop conducted in Canada (2019), to understand the mechanisms in the interaction taking place between the IMO and the Canadian workshop developers and instructors, using the System Theoretic Accident Model and Processes (STAMP). Individual expert interviews are performed, with the main contributors directly involved in developing and conducting the workshop, to evaluate the event’s contribution to improving and specifying the STCW Convention’s training requirements, as referenced in the Polar Code, for seafarers operating in polar waters.

## Introduction

The Arctic region is experiencing extensive growth in commercial shipping activities, while, simultaneously, the sea ice extent is steadily decreasing, enabling extended seasons and voyages in areas previously considered inaccessible for most ships during large periods of the year (Protection of the Arctic Marine Environment [PAME] 2020; Silber and Adams 2019). Shipping in the Arctic Ocean is associated with additional risks, considering the adverse and prevailing climate conditions with the presence of ice, representing great hazards. Ice accretion, caused by sub-zero temperatures and freezing sea spray coming into contact with ships’ surfaces, is the most hazardous form of icing and also the most common (Karahalil et al. 2020). Uncontrolled, sea spray icing is a great hazard in respect of the risk of loss of ship stability and integrity, as well as equipment failure (International Standard Organization [ISO] 2019). Further, floating ice in many forms constitutes an extremely hazardous condition if colliding with a ship in voyage, involving the risk of damage to hull and structure (Ghosh and Rubly 2015). Ship navigation in Arctic waters can be extremely challenging, with a changing landscape of sea ice, draft restrictions in many areas, lack of hydrographic data and detailed surveys, less reliable navigation and satellite communication, and reduced visibility due to fog or darkness for long periods of the year (Hill et al. 2015; Ghosh and Rubly 2015; DNV GL 2015).

In 2017, *The International Code for Ships Operating in Polar Waters* (Polar Code) was adopted by the International Maritime Organization (IMO), applicable to the Arctic and Antarctic Oceans (International Maritime Organization [IMO] 2017). In conjunction with the Polar Code’s implementation, the *International Convention on Standards of Training, Certification and Watchkeeping* (STCW) was amended the following year (2018); new *Regulations on qualifications and certificates for seafarers* were made applicable, providing training requirements for masters, chief mates and officers in charge of a navigational watch on ships with a Polar Ship Certificate (PSC), operating in open and other polar waters (Norwegian Maritime Authorities [NMA] 2018). In this regard, in 2017, the IMO and the Government of Canada (Transport Canada, flag state authority) signed a Memorandum of Understanding, for Canada to provide financial support and expertise in supporting the implementation of the Polar Code; more specifically, it was agreed that Canada would deliver four regional capacity-building “train-the-trainer” workshops. The objectives were to assist flag state authorities and maritime education and training (MET) institutes in enhancing the skills and competence of maritime instructors, to develop competence-based training programs, to update existing ones, and to improve the delivery of the STCW Basic and Advanced training for dedicated personnel on ships operating in polar waters (Offshore Energy 2017; International Maritime Organization [IMO], (n.d.b).

Governance of polar water ship operations is of interest, and various academic disciplines are involved in investigating the efficacy, implications and consequences associated with the implementation of the Polar Code. The enforcement and application of its function-based requirements and how practices evolve are complex matters to study, considering the various stakeholders involved in ship operations in the Arctic and related maritime activities. This paper uses the System Theoretic Accident Model and Processes (STAMP) (Leveson 2011) to model the complexity of contributing factors. Individual expert interviews are performed with the main contributors to the Canadian capacity-building “train-the-trainer” workshop, to evaluate the event’s contribution to specifying and improving the STCW Convention’s training requirements, as referenced in the Polar Code, for seafarers operating in polar waters. The System-Theoretic Process Analysis (STPA) developed from STAMP is used in this assessment of the workshop (See Results).

In the following, the methodological approach of this paper is provided, followed by a summary of the most relevant IMO instruments applicable for international ship operations in the Arctic, before the results of the analysis of the workshop are presented, which are discussed and concluded on at the end of this paper.

## Method

### Literature review

#### Accident models

During accident investigations, the causes leading up to events and their consequences are investigated. This work is conducted by collecting and analyzing available information, enabling reasoning about the causations, often identified as latent conditions and root causes, influence of human errors, technical malfunctions, poor maintenance, or lack of safety culture (Reason 2016). In a developmental trend, earlier accident models addressing safety and high-risk technologies consider linear interactions as those interactions – of one component in the system with one or more components preceding or following it immediately in the sequence of production – that are recognized as leading to predictable and comprehensible event sequences (even during accidents), and the system is functioning as per design (Perrow 1984). On the contrary, system accidents (or normal accidents) involve the unanticipated interaction of multiple failures, identified by the concepts of complex and tightly coupled systems. This complexity is characterized by multi-component systems, with a high level of interaction between the components occurring in non-linear ways. Complex and non-linear interactions will generally be those, not intended in the design, that lead to unexpected event sequences and that are often related to feedback loops introduced to increase efficiency. A change in one component may trigger a new feedback loop, inhibit an existing one or turn a feedback loop into its opposite, and this interactive complexity may help create new categories of accidents and unknown side effects (ibid., 1984).

Another approach to viewing system accidents also focusing attention on high-risk technologies and complex systems. But rather challenging to explain *why accidents occur*, this research explains *why so few serious accidents occur* (SINTEF 2010). The High Reliability Organizations (HROs) approach is that, despite the hazards of complexity and tight couplings which are characteristic of these systems, the continuous management of safe, reliable, and functional high-risk, high-hazard organizations, over periods of time, is achieved by the organization`s flexibility and ability to sufficiently decentralize, to handle the interactive complexity, and at the same time sufficiently centralize, to handle the tight coupling (SINTEF  2010; Sutcliffe 2011). The HRO theory and its key principles are widely applied to achieve minimal errors in high-risk and high-hazard industries, often operating under unpredictable conditions. An explosion of activity has been experienced in application and research associated with the HRO theory, and a growing number of other industries and sectors in society, e.g., health care systems, are showing interest in adopting its principles (e.g., Sutcliffe 2011; Veazie et al. 2019; Cantu et al. 2020).

The HRO theory is, however, also challenged, i.e., claiming a system can be reliable but unsafe or safe but unreliable suggests that safety and reliability are different properties (Leveson 2011).The traditional way of modeling causations, based on a linear approach is questioned, i.e., accidents are viewed in terms of multiple events and active failures sequenced as a forward chain over time, penetrating the defenses-in-depth, based on root causes and failure events (Reason 2016; Leveson 2011). Such event-chain models are viewed as inadequate for analyzing system accidents and systemic risks involving the entire sociotechnical system, deeply influenced by organizational and human factors (Rasmussen 1997). Systemic risks appear, due to the interaction taking place between the multiple stakeholders defining the system and are deeply interconnected, which means they cannot be managed through the actions of a single sector (International Risk Governance Council [IRGC] 2017). Instead, the management of systemic risks requires the involvement of different stakeholders, including governments, industry, academia, and members of civil society, as they are embedded in the larger context of societal, financial, and economic change. In the recognition that accidents occur due to a combination of unexpected conditions – the concurrence of two (or more) events happening at the same time and affecting each other – complex systemic models have gained attention (Hollnagel and Woods 2006). For such models to be useful, they must be able to capture complex and tightly coupled sociotechnical systems that are controlled in an interface between humans and automated processes, driven by advanced technology and software-intensive systems (Leveson et al. 2006).

##### STAMP

One such model, STAMP, with its dynamic approach, provides a modern theoretical foundation for system safety, in which the various stakeholders and NGOs defining a system are identified in hierarchical safety control structures, recognized by its emergent properties (Leveson 2011). The core principle for maintaining system safety is the enforcement of established safety constraints, where causations are recognized in the interaction taking place between the various stakeholders, sectors, and actors making up each level of the system hierarchy (Leveson 2011). STAMP, moreover, models the interaction taking place between system development and system operation, recognizing that safe operations depend on sufficient transfer of information, experience, and knowledge, not only between the levels in the hierarchy but also between system development and system operation; safe operations depend partly on planning, design, and developmental aspects and partly on operational aspects (Leveson 2011). Risk management in STAMP is viewed as a control problem, and unplanned events occur because of inadequate control or lack of enforcement of safety constraints (Leveson 2011). STAMP views the legislators, i.e., the IMO, at the top of the hierarchical system, facilitating and implementing conventions and regulations and constraining governmental bodies, recognized organizations, flag states and classification societies at the levels below. These stakeholders perform controlling activities (e.g., verification, audits, and certifications) at lower levels in the hierarchical system, represented by certified MET institutes, shipowners, and operators, with their management, engineers, and planners and, finally, the personnel assigned on ships operating worldwide. Proper control of established work processes, with adequate constraints ensuring correct behavior, function and interaction in the hierarchical system, is of essence to manage risks in such systems (Leveson 2011; Rasmussen 1997).

#### The Polar Code and Arctic shipping

Empirical data was gathered in document studies covering risk governance of shipping in the Arctic region and the implementation of the Polar Code and the STCW Convention’s training requirements for seafarers operating in polar waters. A survey of scholarly sources was carried out, using databases found under the categories of science and technology, safety, economics, and planning, i.e., Scopus and Web of Science. In addition, cross-checking of the obtained data sources was performed in the multi-discipline database of Google Scholar, combining the search words: “Polar Code”, “STCW”, “Arctic shipping”, “Safety”, “Training”, and “Risk management”.

### Data collection and analysis of the Canadian workshop

#### Interviews

Information was collected through individual interviews with the three persons responsible for developing the Canadian workshop, who also functioned as facilitators and instructors during the event. In the initial design of the study, a request was forwarded to the contact person in the IMO for disclosure of the contact information of the participants in the Canadian workshop, to perform interviews, capturing the participants’ points of view and perceptions regarding the workshop’s objectives and learning outcomes. However, the Legal Division within the IMO stated that names and contact details of participants attending IMO events are confidential information which cannot be disclosed to third parties, for reasons of privacy and the protection of individuals’ integrity. Therefore, the study design was changed to examine the development, conducting, and outcome of the Canadian capacity-building workshop, from the developers and instructors’ point of view. The interviews were performed during July 2020 via telephone, using a prepared interview guide, provided to the interviewees prior to the sessions. This interview guide contained questions concerning the establishment, development, performance, and outcomes of the workshop. The interviews, all lasting approximately one hour, were conducted in a semi-structured manner, allowing flexibility to explore spontaneous issues raised by the interviewees (Ryan et al. 2009). Individual interviews represent the most common data collection strategy in qualitative research (Sandelowski 2002) and were selected as the method, enabling in-depth examination, to capture the experts’ knowledge and understanding of the studied topic (Jacobsen 2015; Labuschagne 2003).

All the interviewees have years of experience in work regulated by the STCW Convention, both prior to and after the Polar Code implementation in 2017 and following the amendments to the STCW in 2018. Interviewee no. 1 has been working within the IMO for more than a decade and is employed as a technical officer in the maritime safety division and the training and human element section. This interviewee works with the STCW Convention and the training and certification of seafarers, including the new STCW Polar Code ice navigation model courses (basic and advanced). In their role as an IMO representative, this interviewee was responsible for overseeing that the workshop was planned and executed according to IMO standards and the objectives set for the event; Interviewee no. 1 also participated in developing the course material and facilitated the workshop as it unfolded. Interviewee no. 2 worked for years at sea (master mariner), mainly in the Arctic Ocean, and possesses years of experience in building and delivering maritime simulation training, including various IMO model courses. This interviewee participated as a representative for Transport Canada in the IMO, with the work of defining the new STCW training requirements that arose from the Polar Code. In connection with this work, the interviewee was engaged to develop and participate as main instructor in the first of four regional capacity-building workshops. Interviewee no. 3 also has years of experience in Arctic shipping (master mariner) and works as the director at the maritime simulation training center where the workshop took place. This interviewee participated in developing the workshop material and facilitated as the event unfolded. The five-day capacity-building “train-the-trainer” workshop was held at a highly technological maritime simulation training center in Canada. This center had previously collaborated with the IMO, due to its extensive experience, stretching back two decades, in developing and providing ice navigation training courses (basic and advanced). These courses were used as basic examples during the development of the current basic and advanced Polar Code ice navigation training courses.

#### Additional data

The interviewee who acted as the main instructor in the workshop provided the handbook “Workshop Materials – Worked Examples” (pp. 71–110), covering the topics: regional regulations, basic operations, passage planning and advanced operations. This workbook contains references to regulatory documents and, for each of the named topics, provides: worked examples of open book exploration questions; job task analysis worksheets; table-top exercises; polar exercise briefing documents; and scenario creation worksheets. Additionally, this interviewee provided a video link (5 min and 34 s) showing a remote-control simulation session that was recorded after workshop completion at the same maritime simulation training center. The objectives of recording this session were to document and present to the IMO how online remote simulation training can be provided to maritime students who do not have access to MET centers providing highly technological simulation, by use of virtual communication systems.

The IMO model courses (6.09) Training course for instructors (International Maritime Organization [IMO] 2010) and (7.11) Basic and Advanced Training Ice Navigation in Polar Waters were used as supporting material in the development of the interview guide. The 6.09 model course material has been designed to identify the basic entry requirements and trainee target group for each course in universally applicable terms, and to clearly specify the technical content and levels of knowledge and skill necessary to meet the technical intent of IMO conventions and related recommendations (International Maritime Organization [IMO] 2010, p. 1).

#### Analysis

In the interviews, this author addressed the following four areas of concern, assumed to be of high criticality for the performance and outcomes of the workshop: (1) competence level of workshop developers, instructors, and participants; (2) suitability of learning plan and methods of teaching; (3) routines for workshop evaluation and documentation (4) and for the exchange of information and learning outcome to MET institutes after workshop completion. Moreover, the collected data has been analyzed utilizing thematic analysis, which is a widely used qualitative analytic method for identifying, analyzing, and reporting patterns and themes in data (Braun and Clarke 2006). Themes were identified using a theoretical approach, providing a detailed analysis of certain aspects of the collected data. The thematic analysis was conducted using the following steps: (1) familiarizing, by transcribing the data; (2) generating initial codes, by exploring features of interesting data across the entire data set; (3) collating the data relevant to each code in a systematic manner; (4) collating codes into potential themes and reviewing these themes, by checking the logical relationship to the coded extracts and the entire data set; (5) defining and naming the themes; and (6) final analysis of selected extracts (Braun and Clarke 2006). Further, STAMP and the related STPA were used as methodologies to assess the workshop (See Results).

## Governance and regulation of international ship operations in the Arctic

Regulating international ship operations is based on a global regulatory regime, built on international maritime conventions, established under the United Nations Convention on the Law of the Sea (UNCLOS). UNCLOS relies on international cooperation between intergovernmental organizations as a mechanism for the development, establishment and implementation of new conventions and regulations. In this regard, “the competent international organization”, as referred to in UNCLOS – being the lead institution to address maritime matters – is interpreted to mean the IMO (Chircop 2017).

### The international maritime organization – IMO

The IMO plays an instrumental role in generating maritime regulations, rules, standards, procedures and recommended practices governing international shipping; it facilitates the national implementation of international instruments, promoting frameworks and practices for cooperation between maritime administrations and the industry (Chircop 2017; Hebbar et al. 2020). The institutional structure of the IMO consists of the Assembly, Council, Secretariat and specialized committees and sub-committees, responsible for keeping the regulatory framework of the IMO developed and maintained on a continuous basis. National delegations drive committee work and formally make decisions, heavily influenced by the participation and involvement of other intergovernmental and non-governmental organizations (NGOs),^1^ encompassing a wide range of associations for industry, maritime labor, environmental protection, education and training, and various professions (Chircop 2017).

### The international convention for the safety of life at sea – SOLAS

The most important of all international treaties concerning the safety of merchant ships is reckoned to be the *International Convention for the Safety of Life at Sea* (SOLAS) (International Maritime Organization [IMO] 2001). The first version was adopted in 1914, in response to the Titanic disaster, later updated and amended on numerous occasions. The main objective of the SOLAS Convention is to specify minimum standards for the construction, equipment, and operation of ships, compatible with their safety.

### The international code for ships operating in polar waters – polar code

**Fig. 1:**
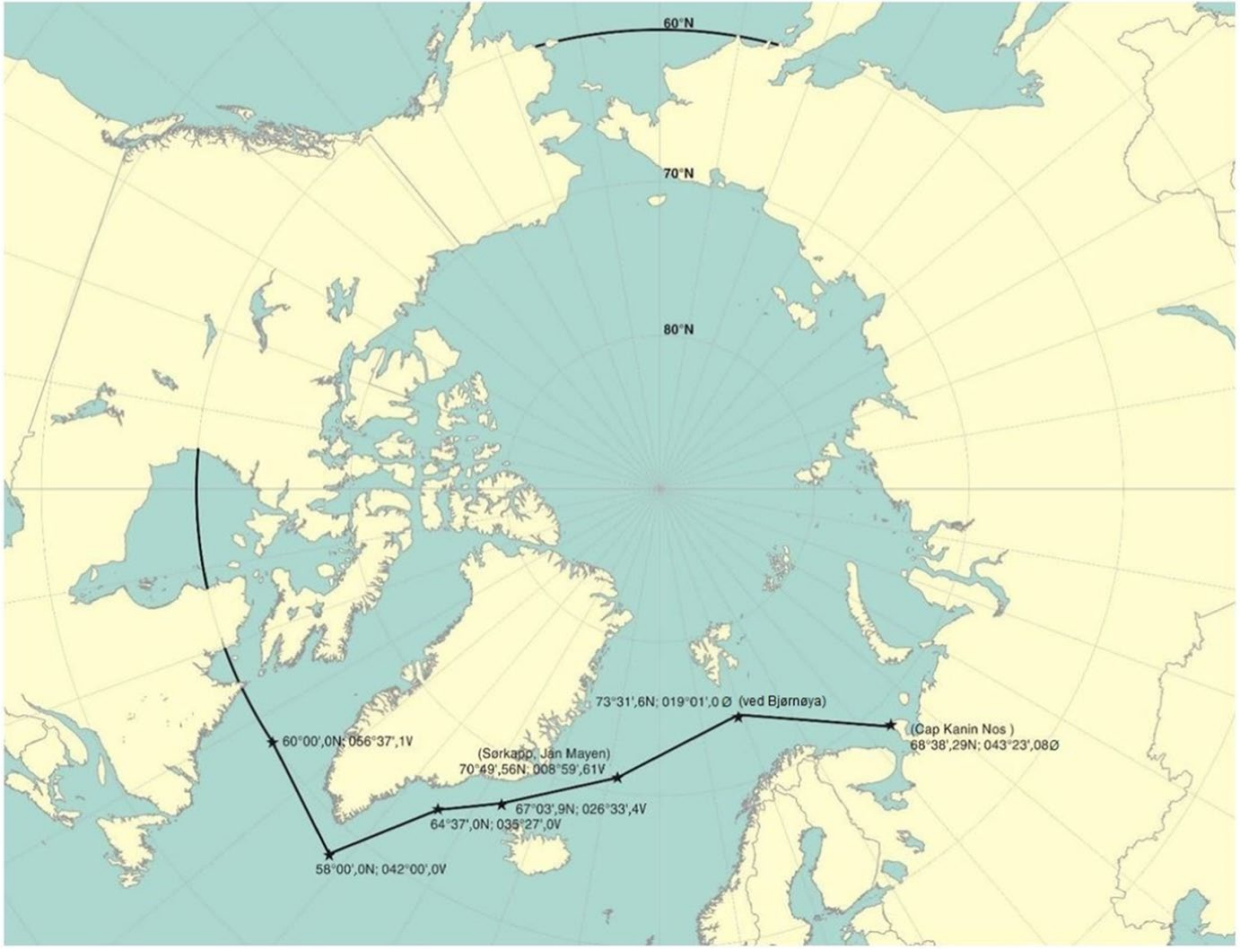
The maximum geographical extent of the Polar Code's area of application in the Arctic (International Maritime Organization [IMO] 2017)

The implementation of the Polar Code was the first international mandatory regulation addressing risks present in polar waters and not adequately mitigated by other instruments of the IMO, regarding the design and construction of ships and equipment, operational conditions, voyage planning, manning, and training, and the protection of the environment (International Maritime Organization [IMO] 2017). The geographical area of application for the Polar Code in the Arctic Ocean is seen below in Fig. 1.

The Polar Code constitutes a continuation of existing regulations, made mandatory under SOLAS, the STCW Convention, and the *International Convention for the Prevention of Pollution from Ships* (MARPOL), applicable to all waters and providing mandatory safety and environmental provisions for ships operating in defined geographical areas around the South and North Poles (International Maritime Organization [IMO] 2017). Ships’ systems and equipment addressed in the Polar Code shall satisfy at least the same performance standards as those referred to in the SOLAS Convention (International Maritime Organization [IMO] 2017), and in this way a standardized minimum of expectations is established for merchant ships for the provision of safety measures for maritime design, equipment, systems, and operations in polar waters.

### The polar code “Toolbox”

The Polar Code is not a stand-alone regulation but must be regarded in the context of the related IMO instruments and other instruments of international law, in addition to applicable national regulations, depending on the areas of operation (Chircop 2017). First, in order to establish procedures and operational limitations for a ship and, accordingly, the issuance of the PSC, an operational assessment of the ship and its equipment is required, taking into account the anticipated range of operating conditions and hazards the ship may encounter in polar waters (International Maritime Organization [IMO] 2017). Ten sources of hazards are listed in the Polar Code that shall be addressed in the operational assessment; of relevance in this paper is the potential lack of ship crew experience in polar operations, with the potential for human error. Additionally, the issuance of the PSC relies on an assessment of the ship’s operational limitations in ice, with reference to methodologies for assessing operational capabilities and limitations in ice and the Polar Operational Limit Assessment Risk Indexing System (POLARIS) (International Maritime Organization [IMO] 2016). Moreover, the Polar Code requires that information on ship-specific capabilities and limitations in relation to the aforementioned operational assessment shall be included in the Polar Water Operational Manual (PWOM), to be carried on board the ship on voyage (International Maritime Organization [IMO] 2017). The purpose of the PWOM is to provide the owner, operator, master, and crew with sufficient information regarding the ship's operational capabilities and limitations, to support their decision-making process.

### The international convention on standards of training, certification and watchkeeping – STCW

Chapter 12 of the Polar Code contains provision on manning and training, with a goal to ensure that ships operating in polar waters are appropriately manned by adequately qualified, trained and experienced personnel. In order to achieve that goal, companies must ensure that masters, chief mates and officers in charge of a navigational watch on board ships with a PSC, operating in polar waters, have completed appropriate training, taking into account the related provisions in the STCW Convention. In conjunction with the work of implementing the Polar Code, in 2016, the Maritime Safety Committee (MSC) of the IMO adopted mandatory minimum requirements for the training and qualifications of masters and deck officers on ships operating in polar waters. These became mandatory under the STCW Convention from 1 July 2018, as *Amendments to the Regulations on qualifications and certificates for seafarers* (Norwegian Maritime Authorities [NMA] 2018).

The 1978 STCW Convention was the first IMO regulation to consider the human element’s contribution to safety at sea, establishing basic requirements for training, certification and watchkeeping for seafarers on an international level (International Maritime Organization [IMO], n.d.a; Hagerupsen 2019). In 1995, the STCW Convention underwent a major revision and was amended in response to a recognized need to bring it up to date and to respond to critics who pointed out vague or unclear requirements, which resulted in different interpretations of the regulation (International Maritime Organization [IMO], n.d.a). The STCW-95 Convention provided more detailed requirements for minimum standards of competencies for seafarers, essentially requiring students to demonstrate their competence to prescribed standards (Ghosh 2017). The focus shifted from being a knowledge-based Convention, comprising a syllabus for qualifying examinations, to providing requirements of skills and abilities necessary to perform workplace tasks (Ghosh 2017).

The STCW Convention has contributed to building the capacity and education of seafarers, creating uniform standards at the global level to supplement national legislation, and introducing relevant rules to enhance professionalism on board vessels, especially in situations where seafarers have been carrying out their jobs on board foreign flag vessels (Munari 2020). Many of the MET institutes use simulators and practical exercises for training and assessment in selected courses, developed to satisfy the STCW Convention, which promotes the use of simulators in MET. However, it is important to consider that a seafarer’s competence is usually demonstrated only in oral or written exams (Castells et al. 2016). The use of decontextualized traditional assessment methods (e.g., multiple-choice questions, pen and paper testing, oral examinations) for most of the units of competence listed in the STCW Convention cannot be ignored (Ghosh 2017). In this regard, the Polar Code has been criticized for being vague in some of its provisions (Todorov 2020) and for not providing sufficient requirements for the manning and training of crews on ships operating in polar waters (Roach 2017). The STCW Convention’s training requirements, implemented in 2018, for dedicated personnel in charge of a navigational watch on ships operating in polar waters, suggest various methods for demonstrating competence, defined as: approved in-service experience, approved ship experience and training, approved simulator training where appropriate, and approved specialist training (Lovdata 2018). However, a lack of harmonization between IMO member states regards the utilization of teaching methods in the training of seafarers is apparent (Castells et al. 2016; Evans et al. 2017).

## Results

**Fig. 2:**
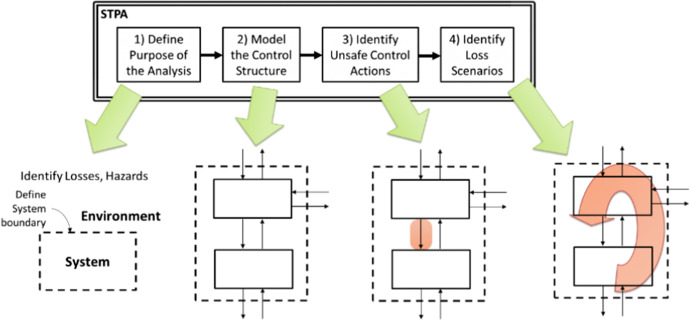
Overview of the basic STPA method (Leveson and Thomas 2018)

The methodology of the STAMP and its related STPA are used in the process of analyzing and assessing the collected data. STPA is a proactive method used to analyze complex processes and the interactions among system components, enabling hazards to be eliminated or controlled before accidents occur. A graphical representation of the four steps in the basic STPA (Leveson and Thomas 2018), which are applied in this analysis, is shown in Fig. 2.

### Phase 1 – Defining the purpose of analysis

The Polar Code is applicable to SOLAS-certified ships operating in the Arctic and Antarctic regions that are passenger ships carrying more than 12 passengers or cargo ships with a gross tonnage of 500 or more, engaged in international voyages (International Maritime Organization [IMO] 2001). The STCW Regulations regarding qualifications and certificates for seafarers and requirements for the completion of appropriate ice navigation training primarily involve training requirements for masters, chief mates, and officers in charge of a navigational watch on ships with PSC operating in open and other polar waters. This training shall be conducted at a MET institute offering an approved test, which shall be documented with an associated Certificate of Proficiency, basic or advanced (NMA 2018).

The IMO’s mission is to promote safe, secure, environmentally sound, efficient, and sustainable shipping through cooperation. This shall be accomplished by adopting the highest practicable standards of maritime safety and security, efficiency of navigation, and prevention and control of pollution from ships, as well as through consideration of the related legal matters and effective implementation of the IMO’s instruments, with a view to their universal and uniform application. The development and conducting of the regional capacity-building “train-the-trainer” workshops is aligned with this mission, aiming to assist in the implementation of the Polar Code and the enhancement of the skills and competence of maritime instructors working at national training institutes. Failing its mission would represent a loss for the IMO, resulting in mismanagement and failure to achieve the objectives established for the workshop. The following four hazards, identified by this author, could compromise the objectives of the workshop, if safety constraints are not established (see Table 1, below).

**Table 1:** Hazards and safety constraints established by this author for the workshop

No	Hazards	Safety constraints
1	Incompetent workshop developers, instructors, and participants	Only competent personnel to attend as workshop developers, instructors, and participants, i.e., check of work background, references, CV, etc
2	Inadequate learning plan methods of teaching	The learning plan and methods of teaching must be validated prior to workshop start-up
3	Inadequate evaluation and documentation of workshop learning outcome	Routines for evaluation and documentation must be in place, i.e., regular debriefs between instructors and participants to be documented
4	Insufficient transmission of information and learning outcome to MET institutes after workshop completion	System verification to ensure certified MET institutes receive and implement recommended methods of teaching established for the Polar Code ice navigation courses

### Phase 2 – Modeling the control structure

**Fig. 3:**
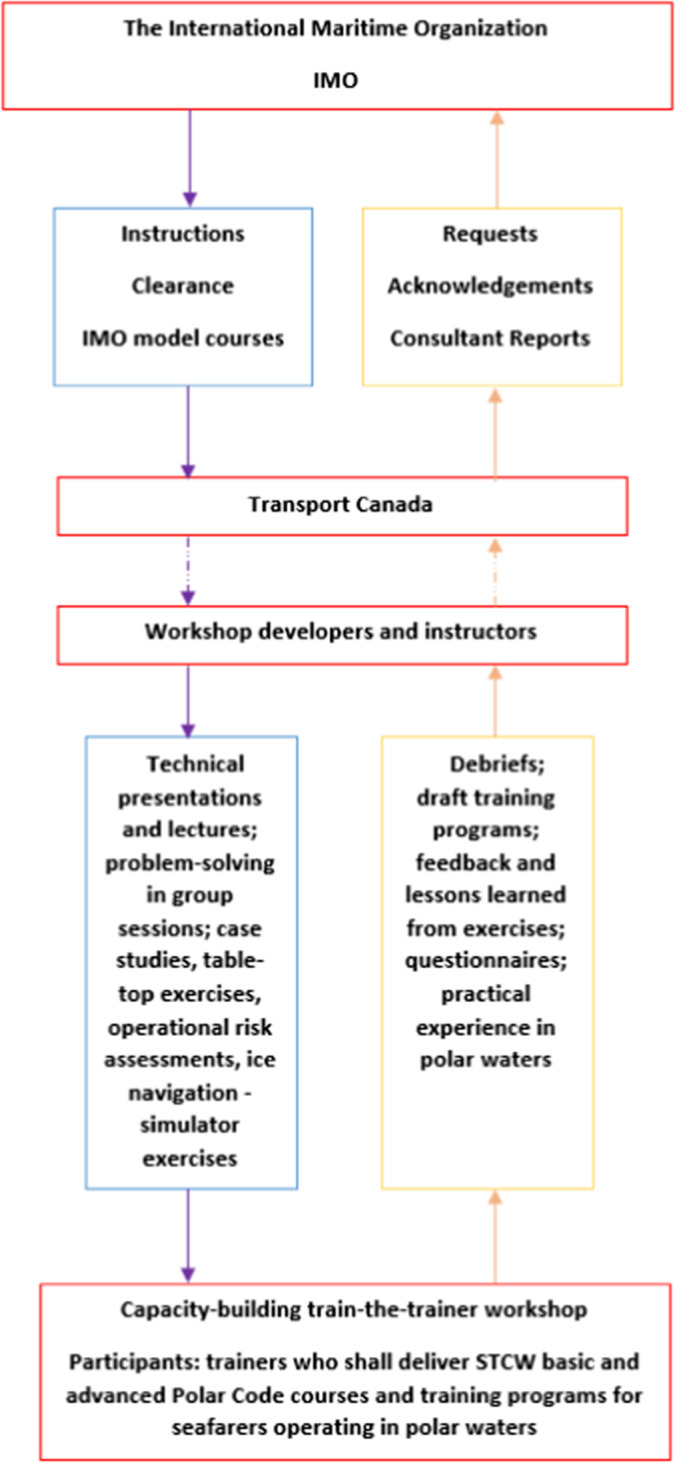
System safety modeling the governance and implementation of the capacity-building “train-the-trainer” workshop ( adapted from Leveson 2011)

The Canadian workshop, modeled in Fig. 3 below, shows the IMO at the top interacting with the Government of Canada, represented by Transport Canada (Canadian flag state authority), previously mentioned as initiating and supporting the implementation of the Polar Code, both financially and by means of expert personnel, in the delivery of four regional capacity-building workshops. At the level below, the interaction between Transport Canada and the persons responsible for developing and conducting the workshop is modeled, where, e.g., instructions, guidelines, and IMO model courses control the development of the workshop, to ensure it meets its objectives. Moreover, the interaction between the workshop’s developers and instructors and its participants is modeled, constrained by, e.g., the training material developed for the event, the competence levels of the instructors and the technological facilities available, i.e., simulators. The participants provide feedback to the instructors regarding the workshop’s progress, in e.g., regularly performed debriefs and questionnaires, and in the delivery of training programs, highly influenced by the participants’ level of competence and experience with polar water operations.

### Phases 3 and 4 – Identification of unsafe control actions and loss scenarios

The term “unsafe” refers to the hazards identified in STPA. These can include issues related to loss of human life or injury (traditional safety), but they can also be defined much more broadly to include other losses, like a mission loss and loss of performance (Leveson and Thomas 2018), as discussed in this paper. After the unsafe control actions are defined,^2^ the loss scenarios can be identified, describing the causal factors that can lead to the unsafe control actions and to hazards, followed by a redefinition of the controlling constraints, summarized in Table 2.

**Table 2:** Summary of hazards, control actions, loss scenarios and controlling constraints for the workshop, identified by this author

No	Hazard	Control action	Not providing causes hazard	Providing causes hazard	Control action provided too early or too late, i.e., at the wrong time or in the wrong sequence	Loss scenario	Redefining controlling constraints
1	Incompetent workshop developers, instructors, and participants	Competence verification of workshop developers, instructors, and participants	Not performing competence verification of workshop developers, instructors, and participants	Inadequate competence verification of workshop developers, instructors, and participants	Competence verification of workshop developers, instructors, and participants not performed in a timely manner	Workshop developed and performed by incompetent personnel and attended by unqualified participants	Verification of routines for competence verification of workshop developers, instructors, and participants
2	Inadequate learning plan and methods of teaching	Quality control of learning plan and methods of teaching	Not performing quality control of learning plan and methods of teaching	Inadequate quality control of learning plan and methods of teaching	Quality control of learning plan and methods of teaching not performed in a timely manner	Learning plan and methods of teaching do not meet the defined objectives for the workshop	Verification of routines for quality control of learning plan and methods of teaching
3	Inadequate evaluation and documentation of workshop learning outcome	Routines for debriefs after training sessions, incl. close-up meetings at the end of the workshop between instructors and participants to be documented	Not performing debriefs and documentation after training sessions, incl. close-up meetings at the end of the workshop between instructors and participants	Insufficient debriefs and documentation performed after training sessions, incl. close-up meetings at the end of the workshop between instructors and participants	Routines for debriefs and documentation after training sessions, incl. close-up meetings at the end of the workshop, between instructors and participants, not performed in a timely manner	Training sessions are not adequately performed, incl. debriefs, and the learning outcome of the workshop is not documented	Verification of routines for debriefs and documentation after training sessions, incl. close-up meetings at the end of the workshop between instructors and participants
4	Insufficient exchange of information and learning outcome with MET institutes after workshop completion	System verification to ensure certified MET institutes receive and implement recommended methods of teaching for the Polar Code ice navigation courses	Not performing system verification to ensure certified MET institutes receive and implement recommended methods of teaching for the Polar Code ice navigation courses	Insufficient verification performed to ensure certified MET institutes receive and implement recommended methods of teaching for the Polar Code ice navigation courses	System verification to ensure certified MET institutes receive and implement recommended methods of teaching for the Polar Code ice navigation courses not performed in a timely manner	Certified MET institutes do not receive and implement recommended methods of teaching for the Polar Code ice navigation courses	Audit of system verification to ensure certified MET institutes receive and implement recommended methods of teaching for the Polar Code ice navigation courses

### Evaluation of the Canadian capacity-building “train-the-trainer” workshop

Only in the last few years has the human dimension of ship operations gained somewhat more international attention; however, the general outlook in international lawmaking remains technical in nature (Kirchner 2018). Although this is also valid for the overwhelming part of the Polar Code, some chapters of the regulation cover the human dimension, e.g., voyage planning (Chapter 11) and manning and training (Chapter 12) (International Maritime Organization [IMO] 2017). The regional capacity-building “train-the-trainer” workshops aim to enhance the skills and competence of maritime instructors, who provide this training, and the qualifications of dedicated personnel in charge of a navigational watch on PCS ships operating in polar waters. These regional workshops are approaching the human dimension, in an interface and combination with technology, using simulator training and practical exercises to address hazards and risks related to voyages and navigation in ice and the associated challenges when working in extreme and harsh polar conditions. 

### Canada’s role in the implementation of the Polar Code and the regional capacity-building “train-the-trainer” workshops

As part of the Polar Code implementation, the Government of Canada provided expertise and financial support, to develop and provide four regional capacity-building “train-the-trainer” workshops, delivering training programs covering the new STCW Convention’s requirements applicable for polar water operations (Offshore Energy 2017). Transport Canada was, according to one of the interviewees, requested by the IMO to provide nominees, who nominated the maritime simulation training center where the Canadian workshop took place. This approach was taken, due to the center’s reputation for delivering world-class simulation technology and industry-driven expertise to solve simulation problems for maritime clientele, one interviewee explained.‌ With years of experience in developing and delivering ice navigation courses, even many years before the implementation of the Polar Code, the center had previously cooperated with the IMO in related matters.

Canada is one of the largest and most politically powerful Arctic states, with a long history of maritime activities in polar waters, mainly consisting of destination traffic to support northern communities and mining industries (Rothwell 2017; Goerlandt and Pelot 2020). Leveraging over 40 years of experience in the oversight of Arctic shipping, Canada played an instrumental role in the development of the Polar Code. This active engagement had great results for the final content of the regulation, which is significantly influenced by Canadian safety and environmental standards (Fraser 2020). From this perspective, the IMO’s approach of engaging Transport Canada to provide financial support and expertise in the deliverance of the regional capacity-building “train-the-trainer” workshops, which were developed and conducted by experts in related matters, is considered by this author as sufficient to ensure the continuation in supporting the implementation of the Polar Code.

### Attendees at the Canadian workshop and requirements for attendance

Eleven participants from seven countries (Canada, The Bahamas, Chile, Denmark, Iceland, India, Jamaica) attended the five-day capacity-building “train-the-trainer” workshop in Canada in 2019, with representatives from flag state authorities and MET institutes. According to one of the interviewees, the aim was to include most IMO member states involved in navigation in polar waters, having seafarers operating in these parts of the world, with the technical cooperation’s department within the IMO choosing relevant countries. Around ten countries were requested to provide two attendees for the workshop, meaning that up to 80 participants potentially could attend if all four regional workshops were conducted.^3^ The Canadian workshop developers were not involved in the selection process of participants, but prerequisites for attendance were forwarded to the IMO. In general, the IMO invites member states to attend IMO seminars, by sending out a profile request for candidates. One interviewee pointed out that not all the countries have delegates with a profile matching these requests and that the candidates nominated are therefore accepted regardless. Based on their previous experiences with the flag states, this interviewee anticipated, in the planning of the workshop, that the prerequisites in many cases would not be used in the selection process of candidates. This interviewee described the selection process as a much more random choice, e.g., a person representing the administration who oversees training and approves a course provider’s submission for Polar Code and STCW Convention ice navigation training can be sent to such an event, to give them briefing and insights into related matters.

The aim and expectation of conducting the workshops – to assist flag state authorities and MET institutes in enhancing the skills and competence of maritime instructors – could be thwarted if many of the attendees are not qualified to attend the events. According to the interviewees, some of the attendees in the Canadian workshop had no experience either with polar water operations and ice navigation or in functioning as certified MET instructors in the subject. The increasing trend in Arctic shipping poses equal challenges and risks of having seafarers used to voyages in e.g., the Mediterranean or Indian Ocean, suddenly finding themselves operating in polar waters, with no previous experience or knowledge about the implications and hazards associated with shipping in these parts of the world. The institutional and individual knowledge concerning risks and hazards related to shipping in the Arctic Ocean remains limited, and even if technology can aid in gathering basic hydrographical information, weather and wave data etc., the human dimension should not be underestimated (Kirchner 2018).

### Methods of teaching used in the Canadian workshop

The new situation of increasing shipping in the Arctic Region requires sufficient preparation of ships and equipment, as well as of the human element, which is where training can be one important aspect of preparing seafarers for polar voyages (Kirchner 2018). The establishment of regional capacity-building “train-the-trainer” workshops, covering the topic of ice navigation in polar waters, aims to standardize this training provided to seafarers operating in the Arctic and Antarctic Oceans. The development and preparation of work documents and material for the Canadian workshop took place during a six-month period, with correspondence and communication going back and forth between the developers, Transport Canada and the IMO, as regards, e.g., objectives, content, learning plan and expected outcomes of the event. Based on previous experience related to flag states affairs, one interviewee explained that it was anticipated that the attendees would be a blend of recently experienced and highly experienced persons, in addition to those with no experience, either as MET instructors or in polar water operations. Therefore, the philosophy guiding the development of the learning plan was that, in a week-long period, a person with zero experience could not reach the level of not only being advanced but able to instruct at an advanced level. The aim became to demonstrate how to develop ice navigation courses, focusing on *what we do, how we do what we do, and how we assess the participants*, as it was expressed by one of the interviewees, and that “*death by PowerPoint presentations*” is not an effective way of delivering these courses.

The main priority was for the various methods of teaching to be useful in the delivery of ice navigation training, rather than focusing on the specific topics and risks associated with ship operations in polar waters. The workshop was structured around practicing skills and competence, with the objective of getting the participants to gain an understanding of the possible achievement, by utilizing practical exercises in the delivery of ice navigation training, even if the requirements in the STCW Convention can be interpreted in terms of a theoretical approach using lectures, as pointed out by one of the interviewees. If the workshop only turned into a lecture piece, with barely any practical elements, it would not be beneficial and in accordance with the objectives established for the event, this interviewee continued. Therefore, more than half of the workshop consisted of exercises or other activities, in which the participants were *doing things*, such as creating lesson plans, designing curriculums, discussing strategies, performing tabletop exercises and risk assessments, with the simulator exercise being the finale part of each session. This approach was selected, this interviewee explained, to get participants to see and experience the merit of not trying to carry out the Polar Code training only through lectures and PowerPoint presentations. Additionally, as pointed out by this interviewee, this approach could highlight weaknesses concerning the STCW Convention’s lack of requirements for practical training in the delivery of courses concerning ice navigation. The existing STCW regimes are questioned in this context, as they mostly define skills required by the seafarers, and there is no guarantee that these skills will prepare seafarers for ship operations in Arctic or Antarctic waters (Kirchner 2018). The development and implementation of practical training requirements for seafarers operating in polar waters should be considered applicable – not only exclusively to masters, chief mates, and officers in charge of a navigational watch but also to the additional crew members assigned to PSC ships (Chaure and Gudmestad 2020).

### Improving Polar Code training through the establishment of regional capacity-building “train-the-trainer” workshops

During the Canadian capacity-building “train-the-trainer” workshop, the participants were engaged to share their opinions during the training sessions, one interviewee explained, in addition to the daily “hot wash” (immediate after-action) debriefs, where the participants shared their experiences. Additionally, the IMO had sessions at the end of the workshop, in which feedback was gathered from the participants through questionnaires. Moreover, this interviewee reported, a daily debrief was conducted between the workshop instructors and the IMO representatives present at the event; these were documented and included in the instructor’s consultant report. The delivery of a consultant report after such an event is a standardized IMO process, this interviewee explained, with a report being produced by the consultant(s) responsible for conducting the event, containing, e.g., descriptions of performed exercises, practices and feedback from the participants.

Participants attending the Canadian workshop desired more practical training and discussions and less lecturing; however, more lectures covering ice recognition were requested. Specifically, participants wanted explicit pointers on how to teach this matter. One interviewee explained that, generally, the ice recognition module does not need to be elaborated in the same manner as was required in the Canadian workshop, where participants had no experience in polar water operations or in functioning as MET instructors. The Canadian workshop was considered a pilot to gain experience, leading to some adjustments before the second workshop was conducted in Chile (November 2019). One of the main improvement points was to provide certain basic knowledge and information for personnel with no experience of polar water operations, as regards ice recognition and identification of the different conditions of ice during the year. These and other findings from the workshop should be taken into consideration going forward, when conducting the remaining workshops, and in future work in developing and establishing new guidelines specifying practical training requirements for seafarers operating in polar waters.

The Polar Code has been criticized for its significant vagueness in some provisions, opening the way for compliance with its functional requirements and overall goals to be interpreted in a variety of ways by administrations and classification societies (Todorov 2020). Further, the practical approach towards training and educating maritime students in ice recognition and identification is a challenging task, especially if the students do not have any experience with cold climate conditions and ice, described as an impossible task by one of the interviewees. Considering that ice and human error are the two main factors of risk occurrence related to ship accidents (collisions, stuck in ice / drift, or sinking and death) in the Arctic, this emphasizes the importance of training and experience in polar water operations (Fedi et al. 2020). The establishment of the regional capacity-building “train-the-trainer” workshops, covering the topic with a practical approach, is therefore considered, by this author, a valuable tool to enhance the competence, skills and knowledge of the instructors who provide the Polar Code training.

### Enhancing the Polar Code training requirements by exchanging information and experiences gathered from the regional capacity-building “train-the-trainer” workshops

After the Canadian capacity-building “train-the-trainer” workshop was conducted, feedback and learning outcomes acquired during the event were included in the consultant’s report and handed over to the IMO. One of the interviewees assumed that this report would only be filed and not used, unless the IMO decided to run a similar workshop at a later stage. According to the interviewees, the task of, and responsibility for, exchanging course material and experiences acquired in the workshop with the respective flag state authorities and MET institutes are placed on the workshop’s participants. This manner of exchanging information was one of the purposes of the workshops, one interviewee explained, assuming that, if course material or other information was distributed by mail only, this would not be prioritized by the recipients. Therefore, the preference for the workshops is to have two attendees representing one member state and, hopefully, as this interviewee expressed it, these participants will bring back and implement what they have learned.

Shipping in polar areas requires special knowledge, skills and experience possessed by a relatively small number of professionals; the lack of clarity in defining the specific skills required of a master and crew operating in polar waters could pose a significant risk to navigation safety (Todorov 2020). Building awareness amongst MET institutes and instructors regards the applicability of implemented policies and regulations is the first step towards effective implementation, and it is of importance that all academic staff of MET institutions are encouraged to have good knowledge of the related matter, for effective training and compliance with the applicable regulations (Evans et al. 2017). The aim of the regional capacity-building “train-the-trainer” workshop – to enhance the skills and competence of MET instructors providing ice navigation training, according to the Polar Code and the STCW Convention – could be jeopardized by relying on non-systematic methods of exchanging experiences and learning outcomes from the events.

## Final Discussion and Conclusion

Adopting and amending international maritime treaties, conventions and regulations is demanding work, and the IMO has been criticized for either not managing to bring new ones into force in a timely manner (Chircop 2017), e.g., the Polar Code’s development and implementation took more than 25 years, or, when in force, not being able to achieve compliance by various stakeholders. In the effort to address this problem, the IMO established the sub-committee on Implementation of IMO Instruments (III) (ibid., 2017), which could be a compatible division within the IMO for addressing concerns regarding the harmonization of the Polar Code and the STCW Convention’s training requirements for seafarers operating in polar waters. However, the primary responsibility for exercising control over ships rests with the flag state authorities. According to UNCLOS (Art. 94), every state shall effectively exercise its jurisdiction and control over ships flying its flag and take necessary measures to ensure safety at sea, regarding, e.g., the construction, equipment and seaworthiness of ships, the manning and labor conditions, and the training of crew members, according to the applicable regulations and requirements in the operating areas (Todorov 2020). Port states may initiate controls and check whether the seafarers’ certificates are valid; whether the manning and qualifications of the crew members comply with the safe manning requirements; and whether the crew is trained according to the Polar Code and the STCW Convention’s requirements for ice navigation in polar waters (Bai and Wang 2019). However, port state controls will be limited and can only determine whether ships have valid documents and whether the standards of the ships and the crew members conform with the information provided in the PSC (Todorov 2020). In addition, the Polar Code does not provide reliable tools to monitor compliance, and the issuing of the PSC is no guarantee that either the ship or the crew’s composition and qualifications are in compliance at any given point in time (Fedi et al. 2018). The control to ensure seaworthiness of ships and their crew members is further complicated by the fact that, if only a few port states carry out strict inspections, the number of ships calling at their port will inevitably decrease, favoring more lax neighbor states (Bai and Wang 2019).

This author considers the Canadian involvement to be adequate, as the first regional capacity-building “train-the-trainer” workshop was developed and conducted by highly competent and experienced persons, with extensive knowledge and skills to teach in the relevant topics. However, some concerns regarding the workshop are raised, namely, some of the attendees’ lack of relevant qualifications and, especially, the delegation to the workshop’s participants, of the responsibility to exchange and transfer experiences and learning outcomes from the event back to national MET institutes and flag states authorities. The first issue, participants who are not qualified to attend the workshop, will limit the goal of reaching out to as many qualified MET ice navigation instructors as possible during these events. Regarding the second issue, delegating the responsibility to the workshop’s participants for the transfer of experiences and learning outcomes back to relevant stakeholders is considered a weak and unreliable way of sharing information within the maritime system. This means of interacting cannot be controlled or assessed in a satisfactory manner, to ensure compliance with the goals established for the workshop. Achieving safety performance in a dynamic system and maintaining a satisfactory safety level over periods of time must be enforced by reliable audits and other reporting tools, ensuring feedback to the system developers about necessary barriers and safety constraints (Hale et al. 2006). In this case, feedback to the legislators was provided after completion of the Canadian capacity-building “train-the-trainer” workshop; however, an inadequacy is pointed out concerning poor control over the transfer and exchange of experiences and outcomes from the legislators and back to relevant stakeholders in the system. Proper information channels and control actions from the IMO to flag state authorities and national MET institutes should be established, to ensure that the majority of relevant stakeholders receive and benefit from the experiences and learning outcomes acquired during the workshops.

In this regard, the STAMP methodology is considered by this author to be a useful tool to identify the maritime system and the stakeholders operating within it. The model helps to explore both established and lack of constraints, affecting the interaction between the various stakeholders participating in the implementation of the Polar Code and the STCW Convention’s training requirements for polar water operations. Safe shipping in these areas relies on stakeholders in the maritime system being aware of and complying with established constraints in a satisfactory manner, in this context awareness of and compliance with applicable regulations and requirements for polar water operations. However, the controls and constraints to ensure that PSC ships are manned with qualified, experienced and skilled personnel for polar voyages are questioned, especially since the responsibility for interpreting the functionally based Polar Code is delegated to the decision makers. The criticism raised by one interviewee, regarding lack of practical training requirements in the STCW Convention, therefore seems legitimate, pointing out that the Polar Code ice navigation courses can be conducted by means of classroom-lectures alone.

## Abbreviations

HRO: High Reliability Organization
IACS: The International Association of Classification Societies
III: (The IMO Sub-Committee on) Implementation of IMO Instruments
IMO: The International Maritime Organization
IRGC: The International Risk Governance Council
ISO: The International Standard Organization
MARPOL: The International Convention for the Prevention of Pollution from Ships
MET: Maritime Education and Training
MSC: The IMO Maritime Safety Committee
NGOs: Non-Governmental Organizations
NMA: Norwegian Maritime Authorities
PAME: Protection of the Arctic Marine Environment
Polar Code: The International Code for Ships Operating in Polar Waters
POLARIS: Polar Operational Limit Assessment Risk Indexing System
PSC: Polar Ship Certificate
PWOM: Polar Water Operation Manual
SOLAS: The International Convention for the Safety of Life at Sea
STAMP: System Theoretic Accident Model and Processes
STCW: The International Convention on Standards of Training, Certification and Watchkeeping
STPA: System-Theoretic Process Analysis
Transport Canada: Canadian Flag State Authority
UNCLOS: The United Nations Convention on the Law of the Sea

## Footnotes

1 During the development of the Polar Code, stakeholders representing various NGOs actively participated in the discourse and deliberations concerning the regulation’s provisions and requirements, e.g., the adoption of the Requirements Concerning Polar Class carried out by the International Association of Classification Societies (IACS), now forming the basis for the Polar Code’s mandatory provisions concerning polar class (Chircop 2017).

2 The failure type, “control action stopped too soon or applied too long” (iv) (Leveson and Thomas 2018), was found not to be relevant and is therefore omitted from the analysis.

3 The currently imposed travel restrictions (COVID-19) have put the implementation of the two remaining workshops, planned to be conducted in the Republic of Korea and the Russian Federation, on hold.

## References

[CR1] Bai J, Wang C (2019). Enhancing port state control in polar waters. Ocean Development & International Law.

[CR2] Braun V, Clarke V (2006). Using thematic analysis in psychology. Qualitative Research in Psychology.

[CR3] Cantu J, Tolk J, Fritts S, Gharehyakheh A (2020). High Reliability Organization (HRO) systematic literature review: Discovery of culture as a foundational hallmark. J Contingencies and Crisis Management.

[CR4] Castells M, Ordás S, Barahona C, Moncunill J, Muyskens C, Hofman W, Cross S, Kondratiev A, Boran-Keshishyan A, Popov A, Skorokhodov S (2016). Model course to revalidate deck officers’ competences using simulators. WMU J Marit Affairs.

[CR5] Chaure MR, Gudmestad OT (2020). Effectiveness of the Polar Code training of cruise liner crew for evacuation in the Arctic and Antarctic. Transnav.

[CR6] Chircop A, Beckmann RC, Henriksen T, Kraabel KD, Molenaar EJ, Roach JA (2017). The IMO, its role under UNCLOS and its Polar Shipping Regulation. Governance of Arctic Shipping, Balancing Rights and Interests of Arctic States and User States.

[CR7] DNV GL (2015) Winterization for cold climate operations (DNVGL-OS-A201). Høvik, Norway. https://rules.dnvgl.com/docs/pdf/dnvgl/os/2015-07/dnvgl-os-a201.pdf

[CR8] Evans UF, Mkpandiok A, Okonna KO (2017). An evaluation of the level of awareness of the STCW-78 as amended in Manila 2010, using maritime education and training institutions as collective compliance mechanism. Australian Journal of Maritime & Ocean Affairs.

[CR9] Fedi L, Faury O, Etienne L (2020). Mapping and analysis of maritime accidents in the Russian Arctic through the lens of the Polar Code and POLARIS system. Marine Policy.

[CR10] Fedi L, Faury O, Gritsenko D (2018). The impact of the Polar Code on risk mitigation in Arctic waters: a “toolbox” for underwriters?. Maritime Policy & Management.

[CR11] Fraser D (2020) A Change in the Ice Regime: Polar Code Implementation in Canada. In: Chircop A, Goerlandt F, Aporta C, Ronald P Governance of Arctic Shipping, Rethinking Risk, Human Impacts and Regulation. Springer Polar Sciences, 10.1007/978-3-030-44975-9 pp. 285–300

[CR12] Ghosh S (2017). Can authentic assessment find its place in seafarer education and training?. Australian Journal of Maritime & Ocean Affairs.

[CR13] Ghosh S, Rubly C (2015). The emergence of Arctic shipping: issues, threats, costs, and risk-mitigating strategies of the Polar Code. Australian Journal of Maritime & Ocean Affairs.

[CR14] Goerlandt F, Pelot R (2020) An Exploratory Application of the International Risk Governance Council’s Risk Governance Framework to Shipping Risks in the Canadian Arctic. In: Chircop A, Goerlandt F, Aporta C, Ronald P. Governance of Arctic Shipping, Rethinking Risk, Human Impacts and Regulation (pp. 15–41). Springer Polar Sciences. 10.1007/978-3-030-44975-9

[CR15] Hagerupsen R (2019) K33: Kontroll av skipets drift og omsorg for personer om bord. MARKOM FS 2020. https://www.marfag.no/k33

[CR16] Hale A, Guldenmund F, Goossens L, Hollnagel E, Woods DD, Leveson N (2006). Auditing Resilience in Risk Control and Safety Management Systems. Resilience Engineering: Concepts and Precepts.

[CR17] Hebbar AA, Schröder-Hinrichs JU, Mejia MQ, Deggim H, Pristrom S (2020). The IMO Regulatory Framework for Arctic Shipping: Risk Perspectives and Goal-Based Pathways. In: Chircop A, Goerlandt F, Aporta C, Ronald P. Governance of Arctic Shipping, Rethinking Risk, Human Impacts and Regulation (pp. 229–247). Springer Polar Sciences. 10.1007/978-3-030-44975-9

[CR18] Hill E, LaNore M, Véronneau S (2015). Northern sea route: an overview of transportation risks, safety, and security. Journal of Transportation Security.

[CR19] Hollnagel E, Woods DD, Hollnagel E, Woods DD, Leveson N (2006). Prologue: Resilience Engineering Concepts. Resilience Engineering: Concepts and Precepts.

[CR20] International Maritime Organization [IMO] (2016) Guidance on methodologies for assessing operational capabilities and limitations in ice (MSC.1/Circ.1519). Geneva

[CR21] International Maritime Organization [IMO] (2017). International Code for Ships Operating in Polar Waters (Polar Code) (MEPC 68/21).

[CR22] International Maritime Organization [IMO] (2001). International Convention for the Safety of Life at Sea (SOLAS) (1974, and 1978 and 1988 Protocol relating thereto: 2000 and 2004 amendments).

[CR23] International Maritime Organization [IMO] (n.d.a). International Convention on Standards of Training, Certification and Watchkeeping for Seafarers (STCW). Geneva, Switzerland. https://www.imo.org/en/OurWork/HumanElement/Pages/STCW-Conv-LINK.aspx

[CR24] International Maritime Organization [IMO]. (2010). IMO model course (6.09) Training course for instructors (2^nd^ edition)

[CR25] International Maritime Organization [IMO]. (n.d.b). *Shipping in Polar Waters.* Geneva, Switzerland. https://www.imo.org/en/MediaCentre/HotTopics/Pages/polar-default.aspx

[CR26] International Risk Governance Council [IRGC] (2017) Introduction to the IRGC Risk Governance Framework, Revised version. Lausanne: EPFL International Risk Governance Center. https://irgc.org/risk-governance/irgc-risk-governance-framework/

[CR27] International Standard Organization [ISO] (2019) Petroleum and Natural Gas Industries — Arctic Offshore Structures (ISO 19906:2019. 2^nd^ ed)

[CR28] Jacobsen DI (2015) Hvordan gjennomføre undersøkelser? Innføring i samfunnsvitenskapelig metode (3. utg. ed.). Oslo: Cappelen Damm akademisk

[CR29] Karahalil M, Ozsoy B, Oktar O (2020) Polar Code application areas in the Arctic. WMU Journal of Maritime Affairs 19, (pp. 219–234). 10.1007/s13437-020-00200-4

[CR30] Kirchner S (2018). The human dimension of the polar code. Australian Journal of Maritime & Ocean Affairs.

[CR31] Labuschagne A (2003) Qualitative research--airy fairy or fundamental? The Qualitative Report 8 (1):100–103. https://nsuworks.nova.edu/cgi/viewcontent.cgi?article=1901&context=tqr

[CR32] Leveson N (2011). Engineering a Safer World: Systems Thinking Applied to Safety.

[CR33] Leveson N, Thomas JP (2018) STPA Handbook (March). https://psas.scripts.mit.edu/home/get_file.php?name=STPA_handbook.pdf.

[CR34] Leveson N, Dulac N, Zipkin D, Cuther-Gershenfeld J, Carrol J, Barret B, Hollnagel E, Woods DD, Leveson N (2006). Engineering resilience into safety-critical systems. Resilience Engineering: Concepts and Precepts.

[CR35] Lovdata (2018) Forskrift om kvalifikasjoner og sertifikater for sjøfolk, Tabell A-V/4–1: Spesifikasjon av minstekrav til kompetanse i grunnleggende opplæring for skip som opererer i polare farvann. https://lovdata.no/dokument/SF/forskrift/2011-12-22-1523/*#KAPITTEL_24

[CR36] Munari F (2020) Search and Rescue at Sea: Do New Challenges Require New Rules? In: Chircop A, Goerlandt F, Aporta C, Ronald P (eds) Governance of Arctic Shipping, Rethinking Risk, Human Impacts and Regulation (pp. 63–81). Springer Polar Sciences 10.1007/978-3-030-44975-9_4

[CR37] Norwegian Maritime Authorities [NMA] (2018) Amendments to the Regulations on Qualifications and Certificates for Seafarers (RSR 05–2018). https://www.sdir.no/en/shipping/legislation/directives/amendments-to-the-regulations-on-qualifications-and-certificates-for-seafarers2/

[CR38] Offshore Energy (2017) IMO: Canada Provides Financial Support for Polar Code. https://www.offshore-energy.biz/imo-canada-provides-financial-support-for-polar-code/

[CR39] Perrow C (1984) Normal Accidents: Living with High Risk Technologies. Princeton University Press

[CR40] Protection of the Arctic Marine Environment [PAME] (2020) The Increase in Arctic Shipping 2013–2019 (Arctic shipping status report (ASSR) #1 31 March 2020). https://www.pame.is/projects/arctic-marine-shipping/arctic-shipping-status-reports/723-arctic-shipping-report-1-the-increase-in-arctic-shipping-2013-2019-pdf-version/file

[CR41] Rasmussen J (1997). Risk management in a dynamic society: a modelling problem. Safety Science.

[CR42] Reason JT (2016). Managing the Risks of Organizational Accidents.

[CR43] Roach AJ, Beckmann RC, Henriksen T, Kraabel KD, Molenaar EJ, Roach JA (2017). The Polar Code and its Adequacy. Governance of Arctic Shipping, Balancing Rights and Interests of Arctic States and User States.

[CR44] Rothwell DR, Beckmann RC, Henriksen T, Kraabel KD, Molenaar EJ, Roach JA (2017). Canada and the United States. Governance of Arctic Shipping, Balancing Rights and Interests of Arctic States and User States.

[CR45] Ryan F, Coughlan M, Cronin P (2009). Interviewing in qualitative research: The one-to-one interview. International Journal of Therapy and Rehabilitation.

[CR46] Sandelowski M (2002). Reembodying qualitative inquiry. Qualitative Health Research.

[CR47] Silber GK, Adams JD (2019). Vessel operations in the Arctic, 2015–2017. Frontiers in Marine Science.

[CR48] SINTEF (2010) Organisational Accidents and Resilient Organisations: Six Perspectives, Revision 2, SINTEF A17034

[CR49] Sutcliffe KM (2011). High reliability organizations (HROs). Best Practice & Research Clinical Anaesthesiology.

[CR50] Todorov A (2020). Coping with deficiencies in the Polar Code: a Russian Perspective. The Polar Journal.

[CR51] Veazie S, Peterson K, Bourne D (2019) Evidence Brief: Implementation of High Reliability Organization Principles. Department of Veterans Affairs, Veterans Health Administration, Health Services Research & Development Service, Washington, DC. 31233295

